# Relationship Between Physical Inactivity and Obesity in the Urban Slums of Lahore

**DOI:** 10.7759/cureus.23719

**Published:** 2022-04-01

**Authors:** Tahseen Kazmi, Luqman F Nagi, Saleem P Iqbal, Shama Razzaq, Shamaila Hassnain, Shehnaz Khan, Noor Shahid

**Affiliations:** 1 Community Medicine, Central Park Medical College, Lahore, PAK; 2 Community Medicine, Shalamar Medical & Dental College, Lahore, PAK; 3 Community Medicine, APPNA Institute of Public Health, Karachi, PAK; 4 Community Medicine, Fatima Memorial Hospital College of Medicine & Dentistry, Lahore, PAK

**Keywords:** survey, physical activity, bmi, obesity, overweight

## Abstract

Introduction

There are several factors such as physical inactivity, sedentary lifestyle, and diet that can be responsible for weight gain or obesity. Regular physical activity is important for better physical and emotional well-being. The objective of the study is to observe the prevalence of obesity or over-weight and how lack of physical activity contributes to weight gain and other health issues.

Methods

This cross-sectional study was conducted in Shalamar Town, Lahore on 646 participants. Data was collected using the WHO STEPS instrument. The inclusion criteria were a minimum age of 30 years and residents of Shalimar Town, Lahore for more than five years. The exclusion criteria were participants with comorbid conditions like HIV, TB, and terminal stage of cancer. Test of association and binary logistic regression analysis was performed to observe a significant association between demographic variables and non-communicable diseases among the participants involved in performing physical exercise.

Results

About 22.1% of the participants had normal BMI, 5.3% were underweight whereas 34.2% of the participants were overweight and 32.4% obese. Male participants were found to be more physically active compared to females. Hypertension and diabetes were statistically significantly associated with physical activity. BMI and waist-hip ratio were found to be associated with moderate physical exercise.

Conclusion

Most of the participants were not involved in moderate physical activity. Overall, an alarming 66.6% of the participants were either overweight or obese. In general, males were found to participate more in intense physical activity.

## Introduction

Physical activity refers to the action that consumes energy and produces skeletal muscle. It is the prime element for the improvement of health [1]. It has a considerable role in fat loss and healthy weight gain. Regular physical activity is important for better physical and mental well-being, and for the prevention of various health issues [2].

Promoting physical activity in the early stages of life is imperative for the healthy growth of children and adults [2,3]. Children and adolescents of age 5-17 years must do moderate to intense physical activity for at least 60 minutes as given in the World Health Organization (WHO) recommendations [3]. Moderate physical activity requires moderate physical effort and causes a small increase in breathing or heart rate or carrying light loads for at least 10 minutes continuously whereas intense physical activity requires hard physical effort and causes a large increase in breathing or heart rate or carrying or lifting heavy loads for at least 10 minutes continuously. Mild physical activity is riding a bicycle or walking for 10 minutes [4].

Increased physical activity has numerous social benefits like community engagement, better social interaction as well as reduced anxiety and depression, increased muscular strength, reduced odds for the development of non-communicable diseases (NCDs), improved respiratory system, strong immune system, improved stamina, and endurance [2,3].

Obesity is related to eating disorders such as binge and night eating disorders [5]. Balanced nutrition together with physical activity leads to a healthy lifestyle that enhances lifelong health [6]. Another global recommendation from the WHO is to do moderate to intense physical activity for 150 min/week to attain and maintain good health [7]. Due to the rapid growth in technology and more scope of social media, physical inactivity has turned into a universal pandemic. Adults mostly prefer to remain sedentary, which makes them more vulnerable to disease or ill health [8].

Chronic health problems and various NCDs are mainly emanated from physical inactivity [9]. The most common and significant health issue associated with physical inactivity is obesity or increased BMI [9]. Physical inactivity and obesity are among the leading risk factors for morbidity and mortality [10]. Obesity is the root cause of many NCDs, like diabetes, hypertension, stroke, and osteoporosis [11].

Weight is maintained by the physical mechanism of a balance between energy expenditure and consumption. When the human body burns fewer calories either because of decreased physical exercise or increased eating, the result is obesity. The final image is of excessive and abnormal fat accumulated in the body [12].

The extent of weight gain varies from factors such as age, gender, ethnicity, etc. [12]. Obesity is said to occur when the body weight exceeds 20% of the ideal weight in respect to a person’s age, weight, and height [12]. Obesity has become the fifth leading risk factor for global deaths. Overweight is the sixth principal risk factor contributing to the overall burden of NCDs [13]. BMI is a measure used to assess obesity. WHO has developed criteria for the global and South Asian populations for obesity [14].

For the global population, BMI ranging from 18.5 to 24.9 is considered normal whereas BMI ranging from 25-29.9 means over-weight, 30-34.9 is obesity class I, 35-39.9 is obesity class II and > 40 is obesity class III. Normal BMI ranges from 18.5 to less than 23 in South Asian countries, 23-27.5 is labeled over-weight and 27.5-32.5, 32.5-37.5, and above 37.5 are obesity class I, II, and III [1].

The objective of the study is to find out the prevalence of obesity and overweight as a result of physical inactivity in the urban slums of Lahore.

## Materials and methods

The study included 646 participants, living in Union Council 120 and 122 of District Lahore, Pakistan. The minimum sample size calculated was 317 using the WHO sample size calculator taking the prevalence of obesity as 29% [1], 95% confidence and 80% power of the study [15]. The data was collected from September 2018 to September 2019. Two-stage sampling was used to collect data. Union Council (UC) was the first stage unit and within each UC, blocking was done as the secondary unit. Samples were selected from a selected block using systematic sampling.

The inclusion criteria were residents living in UC 120 and 122 for more than five years of either sex, with a minimum age of 30 years; exclusion criteria were participants with comorbid conditions like HIV, TB, and terminal stage of cancer. Also excluded were those participants who refused to participate.

The Institutional Review Board of Shalamar Medical College permitted the study. The data was collected from personal interviews using a section of physical activity, physical measurements, and biochemical measurements of the WHO STEPS questionnaire.

The descriptive statistics for the continuous variables were given. Test of association was applied to observe the associated factors with physical activity. Binary logistic regression was performed in light of significant Hosmer-Lemeshow statistics. One-way analysis of variance was performed to observe whether significant differences existed in BMI, blood sugar ratio, waist-hip ratio, heart rate, and blood pressure of the participants who were involved in intense physical exercise. Data analysis was done using SPSS v.26 (IBM Corp., Armonk, NY) [16].

## Results

The average age of the respondents was 44.5 + 13.3 SD (in years). Most of the respondents were female. The proportion of male respondents was 36.8%. Female participants were more obese and overweight (Table 1). Nearly 40% and 22.5% of the male participants were overweight and obese out of the total proportion of overweight and obese participants.

**Table 1:** Gender-wise proportion of BMI (n=646)

BMI	Total	Male	Female
Under-weight	52 (8%)	26 (10.9%)	26 (6.4%)
Normal	164 (25.4%)	77 (32.4%)	87 (21.3%)
Over-weight	221 (34.2)	88 (37%)	133 (32.6%)
Obese	209 (32.4%)	47 (19.7%)	162 (39.7%)

Out of the total sample, 76.5% of the participants performed mild physical activity on a regular basis whereas 22.6% had a routine of doing vigorous to intense physical activity. About 44% were attending moderate physical activities. Male participants were found to be more physically active compared to females. Intense physical activity and gender showed a statistically significant association (Table 2).

**Table 2:** Cross tabulation of physical activity and demographic variables (n=646)

Variables	Categories	Mild Physical Activity			Moderate Physical Activity			Intense Physical Activity		
		Yes	No	p-value	Yes	No	p-value	Yes	No	p-value
Gender	Male	191	47	0.08	105	133	1.00	90	148	0.00*
	Female	303	105		180	228		93	315	
Education-al Level	Illiterate	164	62	0.27	97	129	0.31	58	168	0.22
	Less than Primary	81	31		44	68		29	83	
	Primary	23	06		14	15		12	17	
	Secondary	88	15		41	62		39	64	
	High school	90	25		61	54		28	87	
	College or university	37	09		19	27		13	33	
	Postgraduate	10	04		08	06		04	10	
	Non-response	01	0		01	0		0	01	
Ethnicity	Punjabi	204	67	0.15	109	162	0.23	87	184	0.05*
	Urdu	197	59		122	134		57	199	
	Pushtoon	74	15		43	46		29	60	
	Others	19	11		11	19		10	20	
Marital Status	Never Married	24	10	0.01*	21	13	0.00*	11	23	0.62
	Currently Married	452	128		258	322		166	414	
	Separated	02	0		01	01		0	02	
	Widowed	16	14		05	25		06	24	
Occupation	Government Job	49	09	0.02*	23	35	0.00*	26	32	0.01*
	Private Job	93	16		63	46		37	72	
	Self-employed	35	11		19	27		18	28	
	Student	02	01		02	01		01	02	
	Retired	19	06		06	19		03	22	
	Unemployed (can work)	13	10		03	20		03	20	
	Unemployed (disabled)	08	07		06	09		05	10	
	Household Chores	273	92		162	203		89	276	
	Non-response	02	0		01	01		01	01	

**Figure 1:**
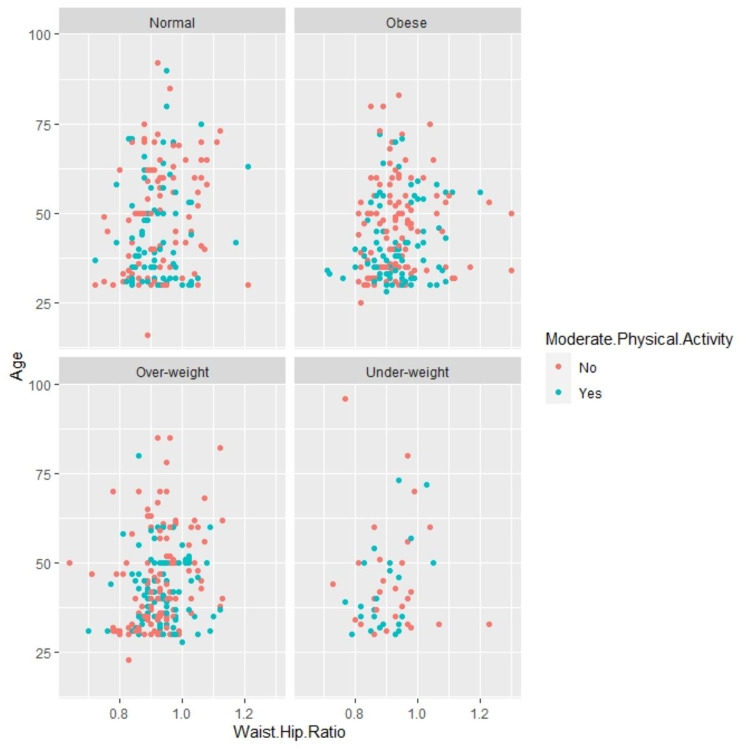
Relationship of various categories of BMI with physical activity

Statistically, significant association was found between mild physical activity and diabetes (p-value=0.000). Hypertension and diabetes were statistically linked with physical activity of moderate nature (p-value=0.008, 0.018). BMI was significantly related to moderate physical exercise (p-value=0.028). Figure 1 illustrates the proportion of participants who fall in various categories of BMI who were involved in moderate physical activity across age and waist-hip ratio.

Hosmer-Lemeshow statistic with a p-value of less than 5% showed that the Binary logistic regression model is a good fit (Table 3). Age was observed as an insignificant factor for intense physical activity. The reference category was male for gender. The reference category for BMI was underweight. Gender, hypertension, stroke or heart attack, and BMI were found as significant factors for intense physical activity. The odds for doing physical activity was 2.008 times higher in males as compared to females. In general, males were found to participate more in intense physical activity. Similarly, participants with a heart attack or stroke were 2.020 times more involved in intense physical activity. However, the negative coefficient of regression for hypertension indicated that odds were 44% lower for hypertensive people.

**Table 3:** Logistic regression analysis to observe factors related with intense physical activity among adults in Lahore (n=646)

Factors	B	S.E.	Sig.	OR	95% C.I. Lower-Upper
Age	0.015	0.011	0.150	1.016	0.994-1.037
Gender (Male)	0.697	0.281	0.013	2.008	1.157-3.484
Raised Cholesterol (Yes)	0.197	0.405	0.627	1.217	0.550-2.693
Hypertension (Yes)	-0.579	0.281	0.039	0.560	0.323-0.972
Diabetes (Yes)	-0.522	0.354	0.141	0.593	0.296-1.188
Stroke (Yes)	0.703	0.330	0.033	2.020	1.058-3.856
BMI (Under-weight)			0.039		
BMI (Normal)	-0.278	0.586	0.634	0.757	0.240-2.385
BMI (Over-weight)	-1.006	0.561	0.073	0.366	0.122-1.099
BMI (Obese)	-1.120	0.566	0.048	0.326	0.108-0.988

## Discussion

Current research projects a clear image of insufficient physical activity in the urban slums of Lahore. The study revealed that 44% of the participants were engaged in moderate physical activity and 22.6% were engaged in intense physical activity. A study reported that 42.8% of participants had a routine of doing a moderate physical activity which is quite close to our finding [17]. Another study in contrast reported 72.6% as the prevalence of physical inactivity [18]. We observed a significant association between physical activity and gender. Comparatively, female participants were more inactive. In a study conducted in Peshawar, Pakistan among undergraduate students, male students were found to be more physically active. The possible reason could be that most females spend their time working at home [19].

The proportion of participants with normal BMI was 25.4% which is quite low in comparison to a study where a proportion of normal BMI was 65.3% [19]. Another research based on the age group 18-65 years also reported the percentage of respondents with a normal BMI of 54.6% [19]. A study conducted in Pakistan in 2020 reported the proportion of participants with a normal BMI as 21% which is very close to our findings [20].

Our study revealed a statistically significant association of BMI with moderate physical activity. Gender and BMI were observed as independently and significantly related to intense physical activity by applying the modal of binary logistic regression. Multivariate logistic regression showed that BMI greater than 33 and age greater than 33 years were significantly independently associated factors for physical activity [19].

A major reason for this public health issue in Pakistan is the lack of awareness [21]. People living in big cities seem to be more exposed to the risk of obesity due to their busy and sedentary lifestyles [21]. Obesity has multiple effects on other NCDs. Pakistan, among the South Asian countries, has the highest percentage of diabetic patients [21]. In the present era, our country Pakistan is in the phase where obesity is directly related to diabetes which is very common irrespective of age, gender, and other socio-demographic characteristics [22-24].

Limitations of the study are the small sample size and the relationship of obesity in the urban slums of Lahore with only one factor, i.e., physical activity. Several other factors such as eating habits, dietary patterns, and other genetic and behavioral factors can also contribute to weight gain and obesity. The association between obesity and these factors must be assessed to observe the contribution of each factor besides physical inactivity.

## Conclusions

The most common physical activity was mild physical activity which was a 10-minute walk among the urban slum dwellers of Lahore. Nearly two-thirds of the participants walked on a regular basis. Less than half of the respondents said that they do moderate physical exercise. Female participants were least involved in moderate and intense physical activity. Overall, 66.6% of the participants were either overweight or obese. BMI and hypertension were significant risk factors for physical inactivity. BMI was significantly associated with moderate physical exercise. In general, males were found to participate more in intense physical activity.

What does our study add?

Obesity is more prevalent among adult females as compared to males in the slum areas of Lahore, Pakistan.

The population-based study showed an alarming rate of participants were either overweight or obese in the urban slums of Lahore, Pakistan.

A significant association was found between mild physical activity and diabetes.

The odds for doing physical activity were two times higher in males compared to females in the slum areas of Lahore.
